# Evaluating the Influence of Chromatic and Luminance Stimuli on SSVEPs from Behind-the-Ears and Occipital Areas

**DOI:** 10.3390/s18020615

**Published:** 2018-02-17

**Authors:** Alan Floriano, Pablo F. Diez, Teodiano Freire Bastos-Filho

**Affiliations:** 1 Postgraduate Program in Electrical Engineering, Federal University of Espirito Santo, Vitoria 29075-910, Brazil; afloriano.ufes@gmail.com; 2 Gabinete de Tecnologia Medica (GATEME), Facultad de Ingenieria, Universidad Nacional de San Juan, San Juan J5400ARL, Argentina; pdiez@gateme.unsj.edu.ar; 3 Consejo Nacional de Investigaciones Científicas y Técnicas (CONICET), San Juan C1425FBQ, Argentina

**Keywords:** SSVEP, visual stimuli, BCI, hairless area

## Abstract

This work presents a study of chromatic and luminance stimuli in low-, medium-, and high-frequency stimulation to evoke steady-state visual evoked potential (SSVEP) in the behind-the-ears area. Twelve healthy subjects participated in this study. The electroencephalogram (EEG) was measured on occipital (Oz) and left and right temporal (TP9 and TP10) areas. The SSVEP was evaluated in terms of amplitude, signal-to-noise ratio (SNR), and detection accuracy using power spectral density analysis (PSDA), canonical correlation analysis (CCA), and temporally local multivariate synchronization index (TMSI) methods. It was found that stimuli based on suitable color and luminance elicited stronger SSVEP in the behind-the-ears area, and that the response of the SSVEP was related to the flickering frequency and the color of the stimuli. Thus, green-red stimulus elicited the highest SSVEP in medium-frequency range, and green-blue stimulus elicited the highest SSVEP in high-frequency range, reaching detection accuracy rates higher than 80%. These findings will aid in the development of more comfortable, accurate and stable BCIs with electrodes positioned on the behind-the-ears (hairless) areas.

## 1. Introduction

The elicited response in the visual cortex by light stimuli flickering at a constant frequency is known as steady-state visual evoked potential (SSVEP) [1]. In the electroencephalogram (EEG), these potentials manifest as an oscillatory component in the signal, with the same frequency (and/or its harmonics) of the visual stimulation [1]. SSVEP can normally be evoked up to 90 Hz [2], and three stimuli bands can be identified: low (up to 12 Hz), medium (12–30 Hz), and high-frequency (≥30 Hz) [3,4,5].

SSVEPs have been used for studies concerning the brain, vision, and the development of brain–computer interfaces (BCIs) [6]. People with severe disabilities can use BCIs as an alternative channel for interaction and communication with the environment around them, only using brain activity [7]. In SSVEP-based BCIs, each stimulus flickers at a specified frequency [8]. Thus, when a person gazes at one of the stimuli, an SSVEP is evoked in the brain [6,9], which can be detected in the EEG signal, and later translated into a control command [10].

Researches have used the SSVEP response to develop assistive technologies, such as robotic wheelchairs [11,12] and robotic exoskeletons [13], as well as for rehabilitation [14,15], communication [16,17], mobile robot control [18], cursor control for computer interaction [4,19], and entertainment [8,20,21].

The SSVEP response is generally maximum on the occipital area of the scalp, and consequently, SSVEPs are strongly detected in the electrodes located at this area [6]. Hence, most of existing SSVEP-based BCIs use electrodes located at O1, O2, and Oz positions. However, this area is generally covered by hair, which causes some complications in the electrode contact with the skin [22,23]. This represents an important drawback in BCI implementation due to the loss of contact between electrode and skin, drying of the gel, especially in long-term operation. In contrast, in hairless regions, it is possible to use different kinds of electrodes, and these drawbacks may be mitigated. Thus, more comfortable BCIs can be designed.

Different studies based on magnetoencephalography (MEG), positron emission tomography (PET), functional magnetic resonance imaging (fMRI), and EEG have demonstrated that SSVEPs can be found in other brain areas, such as parietal, temporal, frontal, and prefrontal areas [9,24,25,26,27,28,29,30]. Thus, using EEG signals from hairless regions to develop a SSVEP-based BCI is a possible option. For example, a system measured SSVEP right in-the-ear using stimulation at low-frequency range (8–11 Hz) [31]. In other work, an electrode was positioned behind-the-ear to acquire the SSVEP [32]. Hsu et al. measured the EEG on the forehead, employing medium-frequency stimuli [33]. In another work, electrodes placed at three hairless areas (behind-the-ears, neck, and face) were used to detect SSVEP [34], in which the authors concluded that electrodes positioned behind-the-ears are the best candidates to build an SSVEP-based BCI in a hairless area. These works used stimuli based only on luminance modulation.

However, visual stimuli that use colors (green-blue or green-red) and luminance combination can increase the evoked response [35,36,37,38,39]. Moreover, it was found that color information is mediated by specialized neurons that are clustered within the temporal areas [40]. Besides, there are color-selective neurons in the inferotemporal cortex [41]. The inferotemporal cortex receives projections from the primary visual cortex (ventral pathways) [42,43], which are both color-sense-associated and object recognition pathways that detect luminance and color. Thus, colored stimuli can enhance the detection of SSVEP in behind-the-ears regions, due to its proximity to the temporal area. Our hypothesis is that chromatic and luminance stimuli can evoke a better response than luminance stimuli in this area.

Therefore, in our work, we present a comparative study of chromatic and luminance stimuli flickering at low-, medium-, and high-frequencies to evoke SSVEP responses in behind-the-ears areas. Basically, this study aims to answer three questions: (1) What is the influence of chromatic and luminance stimuli on SSVEP from behind-the-ears? (2) What is the best combination (green-blue or green-red—note that we did not use the blue-red combination, as these colors are the worst case for photosensitive epilepsy, especially at 15 Hz [44])? (3) How is the SSVEP response evoked by these stimuli in low-, medium-, and high-frequency bands? Therefore, the results of the current work will help in the development of more accurate and comfortable BCI systems.

## 2. Materials and Methods

### 2.1. Data Acquisition

Twelve healthy subjects (ages 26.1 ± 4.1; 6 F and 6 M) with normal or corrected-to-normal vision participated in this study. The EEG recordings were conducted in a laboratory with low background noise and dim luminance. Previous to participation in this study, all volunteers read an information sheet and provided written consent to participate. Ethical approval was granted by the institutional ethics committee. The subjects did not receive any financial reward for their participation.

**Figure 1:**
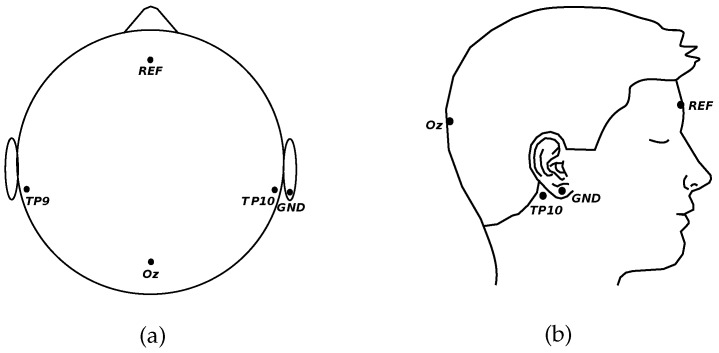
Positions on the scalp where the electrodes were located. (**a**) Top view of positions; (**b**) Side view of the positions. Oz: occipital area; TP9: left temporal area; TP10: right temporal area; REF: reference electrode; GND: ground electrode.

The EEG was measured over occipital (Oz) and left and right temporal (TP9 and TP10) areas (see Figure 1). The ground electrode was placed at A2. The EEG signals were acquired with a Grass 15LT amplifier system, and digitalized with a NI-DAQ-Pad6015 (sampling frequency: 256 Hz). The cut-off frequencies of the analogical pass-band filter were set to 1 and 100 Hz. Additionally, a notch filter for 50 Hz line interference was applied.

### 2.2. Visual Stimulation

The visual stimulation was performed by light-emitting diodes (LEDs) that illuminated a diffusion board of 4 cm × 4 cm. The LEDs were red, blue, green, and white. Each LED could flicker at different frequencies from 5 Hz to 65 Hz with an interval of 5 Hz. Therefore, the stimulation range comprised the three SSVEP bands (low-, medium-, and high-frequency). The frequency of the LEDs was precisely controlled with an Xilinx Spartan3E field-programmable gate array (FPGA) on a Nexys board (Digilent Inc., Pullman, USA). The 50 Hz frequency was not used as a stimulation frequency, because this is the Argentinian power line frequency. The light intensity of the the green and white LEDs was 750 mcd and 250 mcd for blue and red LEDs.

**Figure 2:**
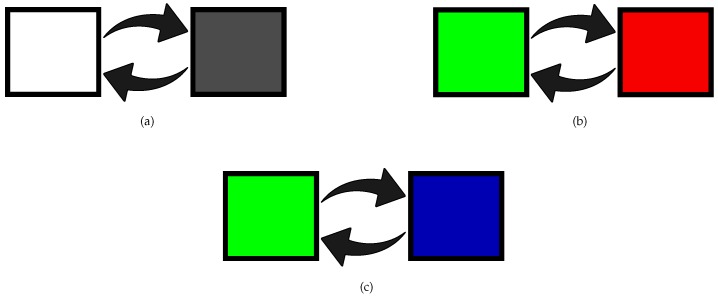
Visual stimulation used for the experiments: (**a**) Luminance stimulus (white, W); (**b**) green-red (G-R) stimulus; (**c**) green-blue (G-B) stimulus.

The setup consisted of three different stimuli (see Figure 2). The first stimulus was white (W) LED for the luminance condition. The W stimulus was configured with 90% of contrast between off and on state, as done by [33]. The other two stimuli were green-red (G-R) stimulus and green-blue (G-B) stimulus for the chromatic and luminance conditions. In this case, the contrast was configured with 50%, such as done by [39]. Figure 2 shows the transition of the two states of the visual stimuli. Each state remained activated for half of the period of the stimulation frequency (f = 1/T, where T is the period). For the luminance stimulus (W), the two states represented the light on and off. For the G-R and G-B stimulus, the two states were green-red and green-blue, respectively.

### 2.3. Experimental Protocol

**Figure 3:**
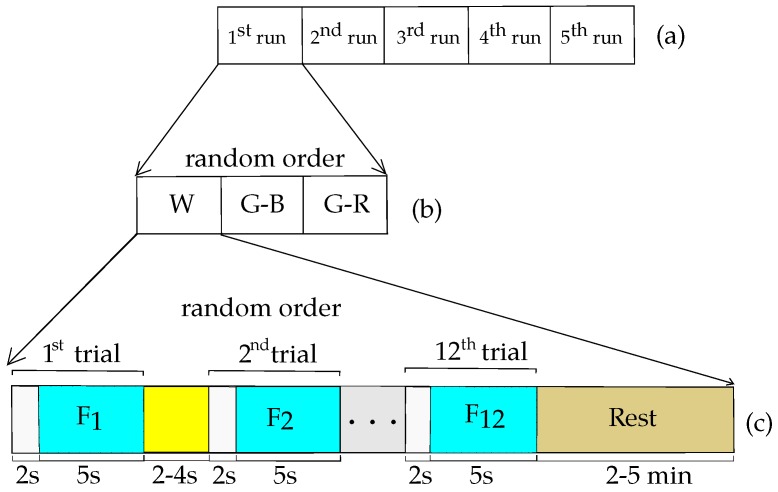
Protocol of the experiment: (**a**) experiment divided into five runs; (**b**) three colored stimuli presented in random order to each volunteer; (**c**) 12 frequencies randomly presented for each colored stimulus.

Each subject sat in a chair at 60 cm from the stimulus. The experiment was divided into five runs (Figure 3a). At each run, the three possible stimuli (G-R, G-B, and W) were showed to the volunteer (Figure 3b). At each colored stimulus, the 12 frequencies were presented. Thus, each stimulus comprised 12 trials, and each trial lasted 7 s (Figure 3c). Thus, 12 trials (one per frequency) of the same colored stimulus were presented to the volunteer. Later, the process was repeated for the other two colored stimuli, which comprised a run. Finally, the run was repeated five times. The stimulation frequencies and the colored stimuli were randomly presented to each volunteer. In order to avoid expectation effects, a variable separation time (2–4 s) between trials was used. The trial began with a beep (at t = 0 s), and 2 s later the stimulus was turned on. The stimulus stayed on until the end of the trial at t = 7 s. At this moment, a feedback was presented to the volunteer indicating whether the SSVEP was detected or not. The volunteer could relax for 2–5 min.

### 2.4. EEG Signal Processing

The EEG was preprocessed using a Butterworth filter, order 6, with cut-off frequencies set at 3 and 70 Hz. Later, the EEG between t = 2 s and t = 7 s was extracted for analyzing in the next step. Then, the magnitude of the frequency components of the signal was calculated based on the discrete Fourier transform (DFT) of the signal x[n] defined as:

(1) $$F\left(f\right)=\left|\sum_{n=0}^{N-1}x\left[n\right]e^{-j2\pi fnT_{s}}\right|,$$

where F(f) is the magnitude of the signal, $T_{s}$ is the sampling period, *N* is the total number of samples of the signal, and *f* is the frequency.

The signal-to-noise ratio (SNR) measurement was computed based on the values extracted from Equation (1). The SNR of SSVEP at a single channel is defined as the ratio of F(f) to the mean amplitude of the *K* neighboring frequencies [34,45]:

(2) $$SNR=\frac{K\times F(f)}{\sum_{n=1}^{K/2}\left[F\left(f+n\Delta f\right)+F\left(f-n\Delta f\right)\right]},$$

where Δf is the frequency resolution (0.2 Hz in this study), and *K* was set to 8 (i.e., four frequencies on each side) [46].

### 2.5. Statistical Evaluation

For the statistical analysis of the results, the Friedman test for simultaneous comparison of more than two groups was used. Post-hoc pairwise comparisons using Wilcoxon signed-rank test were also conducted, in which a level of p< 0.05 was selected as the threshold for statistical significance.

## 3. Results

This section is divided in three parts, where results about amplitude, SNR, and a simulation of SSVEP classification are presented.

### 3.1. Amplitude

**Figure 4:**
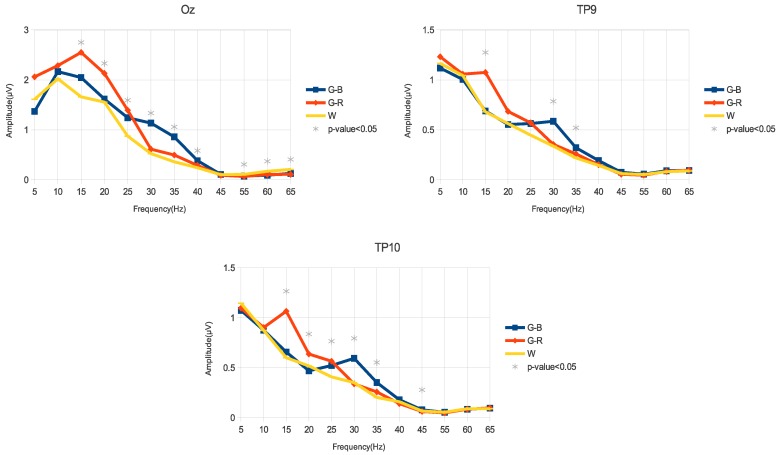
Average of the steady-state visual evoked potential (SSVEP) amplitudes of all volunteers for the Oz, TP9, and TP10 channels using three different stimuli. The frequencies with statistical significance (*p*-value < 0.05) based on the Friedman test are marked with an asterisk.

Figure 4 shows the average amplitudes of the elicited SSVEP of all volunteers for the three visual stimuli. The frequencies marked with an asterisk show statistical significance (*p*-value <0.05) using the Friedman test. The amplitudes were calculated according to Equation (1). Then, for each volunteer, the amplitude value was obtained at each frequency F(f), and the average was computed across the volunteers.

At the occipital region, the G-R stimulus showed a higher response when compared with the W stimulus in the medium-frequency range (15–25 Hz, with *p*-value < 0.05). In contrast, in the high-frequency range (30–40 Hz, *p*-value < 0.05) the G-B stimulus presented a better response when compared with the W stimulus. In the 55–65 Hz interval, the W stimulus achieved a better response than G-R and G-B stimuli.

In the temporal region (TP9 and TP10), a similar behavior was observed; i.e., in the medium-frequency range, the G-R stimulus achieved higher amplitudes (TP9: 15 Hz, with *p*-value < 0.05; TP10: 15–25 Hz, with *p*-value <0.05) than W and G-B stimuli. In the high-frequency range, the G-B stimulus showed a better response (30–35 Hz, with *p*-value <0.05) than the other stimuli.

### 3.2. SNR

**Figure 5:**
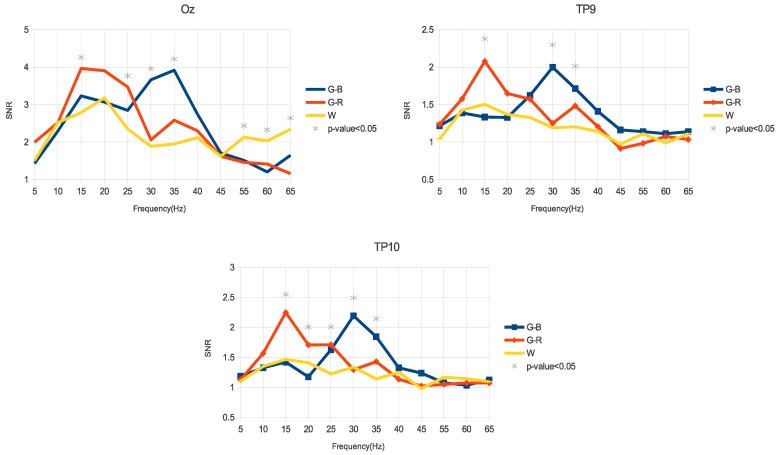
Average of the SSVEP SNR of all volunteers for the Oz, TP9, and TP10 channels using the three different stimulus configurations. The frequencies with statistical significance (*p*-value <0.05) based on the Friedman test are marked with an asterisk.

Figure 5 shows the average SNR of the SSVEP of all volunteers for the three stimuli. The frequencies marked with an asterisk show statistical significance (*p*-value < 0.05) using the Friedman test.

At the occipital region, the G-R stimulus showed a higher response than the W stimulus in the medium-frequency range (15–25 Hz). In the high-frequency range (30–40 Hz), the G-B stimulus showed a better response (30–35 Hz, *p*-value <0.05) than the W stimulus. In the 55–65 Hz range, the luminance stimulus (W) presented a better response than the G-R and G-B stimuli.

Again, a similar behavior was observed in the temporal region (TP9 and TP10); i.e., in the medium-frequency range, there was a higher response of the G-R stimulus (TP9: 15 Hz, with *p*-value < 0.05; TP10: 15–25 Hz, with *p*-value < 0.05) compared with the luminance stimulus (W). In the high-frequency range the G-B stimulus showed a better response (30–35 Hz, with *p*-value <0.05) when compared with the W stimulus.

### 3.3. Simulated SSVEP Classification

Aiming to provide an overview of how the different SNR of SSVEP would impact the design of a future BCI system, a simulated online analysis was performed. Hence, the accuracy of the SSVEP detection [47,48] and information transfer rate (ITR) [9] were used. To emulate an online detection process, the EEG signal was segmented in 4 s windows (as done by [34]). Then, power spectral density analysis (PSDA) [45], canonical correlation analysis (CCA) [49] and temporally local multivariate synchronization index (TMSI) [50] methods, with criterion of maximum value were used for data classification. For this test, two frequencies were chosen in order to simulate a binary BCI. Thus, 15 and 20 Hz were chosen within the medium-frequency range, and 30 and 35 Hz were chosen for the high-frequency range, as these frequencies presented the best SNR (see Figure 5).

**Figure 6:**
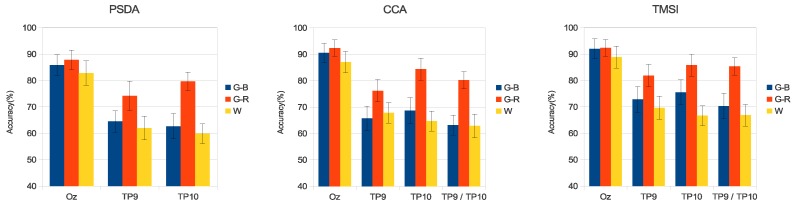
Average accuracy of all volunteers for the three stimuli in medium-frequency range. Error bars indicate standard errors. CCA: canonical correlation analysis; TMSI: temporally local multivariate synchronization index.

**Figure 7:**
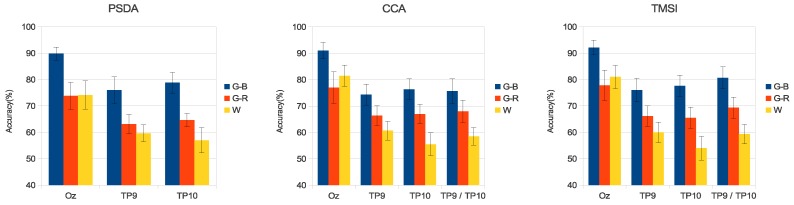
Average accuracy of all volunteers for the three stimuli in high-frequency range. Error bars indicate standard errors.

Figure 6 and Figure 7 present the average accuracy of the classification of all volunteers for the three stimuli in medium- and high-frequency ranges.

**Figure 8:**
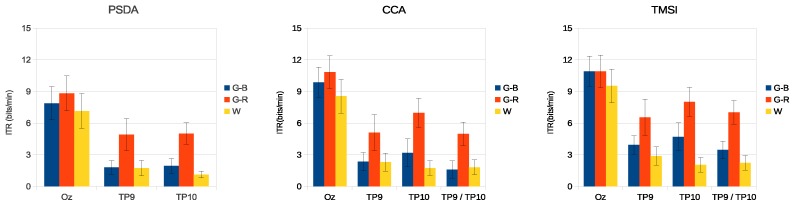
Average information transfer rate (ITR) of all volunteers for the three stimuli for the medium-frequency range. Error bars indicate standard errors.

**Figure 9:**
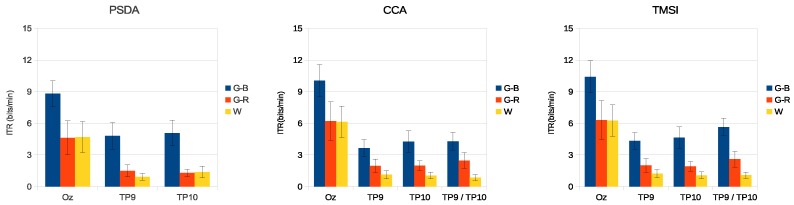
Average ITR of all volunteers for the three stimuli for the high-frequency range. Error bars indicate standard errors.

Figure 8 and Figure 9 present the average ITR of all volunteers for the three stimuli in medium- and high-frequency ranges.

## 4. Discussion

The literature reports that the visual stimuli that combine colors and luminance can increase the evoked response of P300 potentials [35,36]. Similarly, it was demonstrated that the combination of G-B and luminance can evoke a better response in the SSVEP from the occipital region [37,38]. In other work [39], G-R stimuli combined with luminance changes obtained a better response at a modulated frequency of 15 Hz. These works measured the EEG on occipital and parietal regions of the scalp.

Currently, the BCI community is looking at how to transfer these systems from the lab to the patient’s home. Thus, more accurate and comfortable BCI systems must be designed. This way, measuring the EEG from hairless positions presents advantages to the user, and recently, these kinds of BCI systems have been reported in the literature [31,32,34]. These studies demonstrated that it is possible to develop a BCI based on EEG measured from hairless regions; however, concerning the wide frequency range and types of stimulation (color and luminance), the question about the best frequency and type of stimulation remains unclear.

In the current work, SSVEP from behind-the-ears areas (TP9 and TP10) was elicited by three stimuli (G-B, G-R, and W) flickering at low-, medium-, and high-frequency. The aim of this work was to analyze how these stimuli influence the SSVEP response from the behind-the-ears areas. Higher amplitude (Figure 4) and SNR (Figure 5) of the SSVEP were observed when stimuli that combined color and luminance (G-R and G-B) were applied. Particularly, the best response in the medium-frequency band (15–25 Hz) was obtained with G-R stimulation. On the other hand, G-B stimulation showed the best response in the high-frequency range (30–40 Hz). Therefore, a suitable color and luminance stimulation allows the achievement of higher amplitudes and higher SNR from behind-the-ears areas, and consequently, an accurate and comfortable BCI may be designed.

At the occipital electrode, G-R presented the best response in the medium-frequency range, in accordance with [39]. In the current work, the Oz electrode was used as the standard measure in SSVEP and to corroborate the results with TP9 and TP10 electrodes.

The results of the simulated online analysis showed that the combination of colors and luminance also improved the SSVEP detection accuracy and ITR. When stimuli in the medium-frequency range were used, G-R presented better results (see Figure 6 and Figure 8). Using PSDA in TP9 and TP10, the detection accuracy was increased by 12% and 19%, respectively, in comparison with the luminance stimulus (W). Similar behavior was observed with CCA and TMSI methods, with G-R stimulus obtaining better results than other stimuli. CCA and TMSI achieved higher accuracy detection values than PSDA. Specifically, CCA achieved at Oz: 92.3±3.0%, at TP9: 76.1±4.8%, at TP10: 84.3±3.7%, and at TP9/TP10: 80.2±2.8%. On the other hand, TMSI achieved at Oz: 92.3±3.2%, at TP9: 81.8±4.2%, at TP10: 85.8±4.2%, and at TP9/TP10: 85.3±3.3%. As a counterpart, the 15–25 Hz stimulation range can provoke epileptic seizures [1,51].

The high-frequency band is known for its low-amplitude SSVEP, making difficult to implement a BCI [52]. However, when using G-B stimuli, the detection accuracy and ITR were increased on both the occipital and temporal regions (see Figure 7 and Figure 9). The G-B stimulus presented the higher accuracy when compared with G-R and W stimuli using PSDA (Oz: 89.8±2.5%, TP9: 76.0±5.0% TP10: 78.8±4.0%), CCA (Oz: 91.0±3.1%, TP9: 74.3±4.0%, TP10: 76.3±4.0%, TP9/TP10: 75.7±4.6%), and TMSI (Oz: 92.2±2.8%, TP9: 76.0±4.5%, TP10: 77.7±4.0%, TP9/TP10: 80.7±4.2%). In addition, a previous work [44] reports that the green-blue chromatic flicker is the safest stimulus for human visual photosensitivity.

Moreover, the high-frequency stimulation band can provide a reduction of epileptic seizures [51], false positives due to alpha rhythm (8–13 Hz) [1,53], migraine headaches [54], and visual fatigue [54]. Hence, the development of more comfortable and stable BCIs are possible [53]. In the current work, we showed that using G-B stimulus at high frequencies allowed an accuracy rate close to 80% to be obtained in a hairless area.

## 5. Conclusions

This work presented a study of chromatic and luminance stimuli to evoke SSVEPs on behind-the-ears areas. The SSVEP was elicited at low-, medium-, and high-frequency stimulation, and the EEG was measured at left and right temporal (TP9 and TP10) areas. It was found that stimuli based on a suitable color and luminance elicited stronger SSVEP on the behind-the-ears areas. Interestingly, we found a different response of SSVEP related to frequency and color of the stimuli. G-R stimulus elicited the highest SSVEP in the medium-frequency range (15–25 Hz), and G-B stimulus elicited the highest SSVEP at high-frequencies (30–40 Hz). Moreover, detection accuracies of around 80% using PSDA and even more than 80% using CCA or TMSI on a hairless area were obtained. These findings allow the development of more comfortable, accurate, and stable BCIs with electrodes positioned on the behind-the-ears (hairless) areas.

## 6. Future Works

Currently, we are developing an online BCI based on the results of this research to command a robotic wheelchair. Other possible applications for this BCI are spellers for communication, command of telepresence robots, command of autonomous car or for turning on/off home appliances in domotics, and other applications required by people with severe disabilities. A recent work of Chien et al. applied a new paradigm to elicit SSVEPs with little or no flickering sensation and using color stimuli on a screen [48]. However, that work did not use its paradigm on non-hair positions, which can be evaluated using our methodology.

## Abbreviations

The following abbreviations are used in this manuscript:

SSVEP	Steady-State Visual Evoked Potential
BCI	Brain-Computer Interface
SNR	Signal-to-Noise Ratio
REF	Reference Electrode
GND	Ground Electrode
G-R	Green-Red Stimulus
G-B	Green-Blue Stimulus
W	White Stimulus
